# A comparative efficacy study of diagnostic digital breast tomosynthesis and digital mammography in BI-RADS 4 breast cancer diagnosis

**DOI:** 10.1016/j.ejrad.2022.110361

**Published:** 2022-05-17

**Authors:** Chika F. Ezeana, Mamta Puppala, Lin Wang, Jenny C. Chang, Stephen T.C. Wong

**Affiliations:** a Dept. of Systems Medicine and Bioengineering, Houston Methodist Cancer Center, Houston Methodist Hospital, Houston, TX, USA; b Houston Methodist Cancer Center, Houston Methodist Hospital, Houston, TX, USA; c Department of Radiology, Houston Methodist Hospital, Houston, TX, USA

**Keywords:** BI-RADS4-assessed patients, digital mammography, digital breast tomosynthesis, cancer detection rate, biopsy-derived positive predictice value, false-positive biopsies

## Abstract

**Purpose:**

Probability of malignancy for BI-RADS 4-designated breast lesions ranges from 2% to 95%, contributing to high false-positive biopsy rates. We compare clinical performance of digital breast tomosynthesis (DBT) versus digital mammography (2D) among our BI-RADS 4 population without prior history of breast cancer.

**Methods:**

We extracted retrospective data i.e., clinical, mammogram reports, and biopsy data, from electronic medical records across Houston Methodist’s nine hospitals for patients who underwent diagnostic examinations using both modalities (02/01/2015 – 09/30/2020). 2D and DBT cohorts were not intra-individual matched, and there was no direct mammogram evaluation. Using Student’s *t* test, Fisher’s exact test, and Chi-squared test, we evaluated the data to determine statistical significance of differences between modalities in BI-RADS 4 cases. We calculated adjusted odds-ratio between modalities for cancer detection rate (CDR) and biopsy-derived positive predictive value (PPV3).

**Results:**

There were 6,356 encounters (6,020 patients) in 2D and 5,896 encounters (5,637 patients) in DBT assessed as BI-RADS 4. Using Fisher’s exact test, DBT mammography cases were significantly assessed as BI-RADS 4 5.66% more often than those undergoing 2D mammography, *P = 0.0046* (1.0566 95% CI: 1.0169–1.0977). The CDRs were 112.65 (2D) and 120.76 (DBT), adjusted odds-ratio: 1.04 (0.93, 1.16)), *P = 0.5029*, while PPV3 were 14.41% (2D) and 15.99% (DBT), adjusted odds-ratio: 1.09 (0.97, 1.22), *P = 0.1483*; both logistic regression-adjusted for all other factors.

**Conclusion:**

DBT did not achieve better performance and sensitivity in assigning BI-RADS 4 cases compared with 2D, showed no significant advantage in CDR and PPV3, and does not reduce false-positive biopsies among BI-RADS 4-assessed patients.

## 1. Introduction

Mammography plays a vital role in early breast cancer detection and diagnosis [1,2]. It has also been observed that race and socioeconomic determinants are important factors in breast cancer detection, including access to mammography, incidence, and prognosis. Caucasian women are more likely to develop breast cancer than other races (Blacks, Hispanics, and Asians) and are also expected to have the most mammograms performed while breast cancer mortality rates and prognosis are poorer among Black women [3]. Some of these differences in outcomes may be attributed to less access to mammography and lower quality medical care, as well as various lifestyle patterns associated with different ethnic groups [3-5].

The American College of Radiology developed the Breast Imaging Reporting and Data System (BI-RADS) lexicon [6-8] to standardize breast imaging reporting, evaluate risk of breast lesions, and facilitate biopsy decision-making. BI-RADS classifies lesions into seven assessment categories (zero to six), each implying specific management recommendations. However, significant intra- and inter-observer variability from the BI-RADS lexicon has resulted in considerable variation in the rate of biopsy across the US, with 55%–85% of breast biopsies ultimately found to be benign lesions [9,10]. Some other studies have posited that of more than a million biopsies for breast lesions in the US annually, as many as 75% turn out to be benign [11,12]. Among the BI-RADS categories, BI-RADS 4 – “suspicious findings with a recommendation for biopsy” [13], stands out for its enormous uncertainty, with a 2–95% likelihood of malignancy [8,14,15]. A biopsy is thus considered as the most appropriate next course of action for BI-RADS 4 categorized lesions and serves as a quality metric and performance standard [10,16-18] resulting in a majority (69–95%) of BI-RADS 4 lesions being biopsied [19]. Over the decades, BI-RADS 4 tissue biopsy-proven positive predictive value (PPV3) [20,21] rates have not improved [8], and this translates to high false-positive rates of mammography. Currently, BI-RADS 4 PPV3 in the US is reported to be at 21.1% [8]. Researchers have estimated that false-positive mammograms and breast cancer overdiagnoses in the United States cost approximately $4 billion per year [22].

Digital mammograms have been found to show up to 89.3% diagnostic accuracy at detecting breast cancer cases [23], but none of the various standard techniques used for breast imaging are 100% effective, especially for tiny tumors, diffusely infiltrating carcinomas like invasive lobular cancers, and ductal in-situ cancers without microcalcifications [24,25]. With advances in technology, digital breast tomosynthesis (DBT) compared to digital mammography (2D) has been touted to improve cancer detection rates and avoid unnecessary biopsies, despite its slightly higher radiation dose [26-28]. While a lot of hospitals and health systems have transitioned fully to systemwide use of the DBT modality for all mammography examinations, several scientific questions remain unanswered. Some screening (prospective and retrospective) studies projected that a combination of 2D and DBT mammography improves cancer detection compared with stand-alone 2D digital mammography, but with conflicting recall rates [29-35]. However, this combination translates to the risk of more radiation exposure for patients, increased time for interpretation by radiologists, and problems of more significant data storage and management [36,37]. Furthermore, a published study suggested that there may not be much significant advantage in terms of cancer detection rates (CDR), but their DBT (2D + DBT) group had a higher proportion of invasive cancers than in-situ cancers [38].

In this study, we considered diagnostic mammogram examinations given an assessment of BI-RADS category 4 of patients with no history of prior breast cancers in our multi-hospital system over a period of five and half years to compare the clinical performance of DBT mammography i.e., using DBT tomograms alone with 2D mammography, and examined if the DBT mammography confers advantage in terms of better CDR and PPV3.

## 2. Materials and methods

### 2.1. Study population

Our retrospective study was conducted on mammography data extracted from METEOR (Methodist Environment for Translational Enhancement and Outcomes Research), an enterprise-wide clinical data warehouse and analytics environment at our institution [39]. We compared the mammography data for patients who underwent diagnostic (performed based on signs of possible breast cancer or as a further evaluation of suspicious findings from a screening imaging) examinations using 2D digital mammography with that of those who had DBT mammography, performed between February 1, 2015, and September 30, 2020, focusing on those who were given an initial assessment of BI-RADS category 4. They also did not have history of breast cancer to avoid affecting their BI-RADS 4 designation and possible cancer diagnosis. Our hospital’s Institutional Review Board (IRB) approved the study protocol and granted a waiver of written informed consent.

### 2.2. Data collection

METEOR recorded the patients who underwent 2D and DBT as two different patient cohorts. The mammograms were performed using Hologic Lorad Selenia units for 2D, while Hologic Selenia Dimensions and GE Senographe Essential Tomosynthesis units were used for DBT at the Breast Centers throughout our hospital system. The following information was extracted from METEOR: patient demographics including age, gender, race; modality used (2D vs. DBT); presence of prior mammogram; final BI-RADS assessment category; biopsy type: image-guided core needle or surgical biopsy; pathology results within three months after the mammogram; tumor staging; and hormone receptor and growth-promoting protein expression, i.e., estrogen receptor (ER), progesterone receptor (PR), and growth-promoting protein (HER2) status. Available data on tumor staging as well as hormone receptor and growth-promoting protein status was limited, as not everyone had these records. Biopsy outcomes, i.e., benign or malignant lesions, determined by a review of pathology reports, served as cancer diagnosis status. There was no direct mammogram evaluation and the 2D and DBT cohorts were not intraindividual matched.

### 2.3. Statistical methods

R statistical software [40] was used to analyze and compare the data between 2D and DBT. We determined the number of patients among the DBT population who had an intial 2D digital mammography and then subsequent DBT (2D + DBT) examinations. Using student’s t, Fisher’s exact, and Chi-squared tests, we evaluated the collected data to determine statistical significance. Using Fisher’s exact test, we determined the difference in BI-RADS 4 cases and the malignant diagnosis ratio in the BI-RADS 4 biopsy results. We adjusted for confounders such as prior mammograms, age, race, etc., and calculated the adjusted odds ratios between modalities for cancer detection rate (CDR) and biopsy-derived positive predictive value (PPV3). Overall, we tested if there was any significant difference between 2D and DBT mammography in BI-RADS 4 cases. A p-value of <0.05 was considered statistically significant.

## 3. Results

**Fig. 1.**
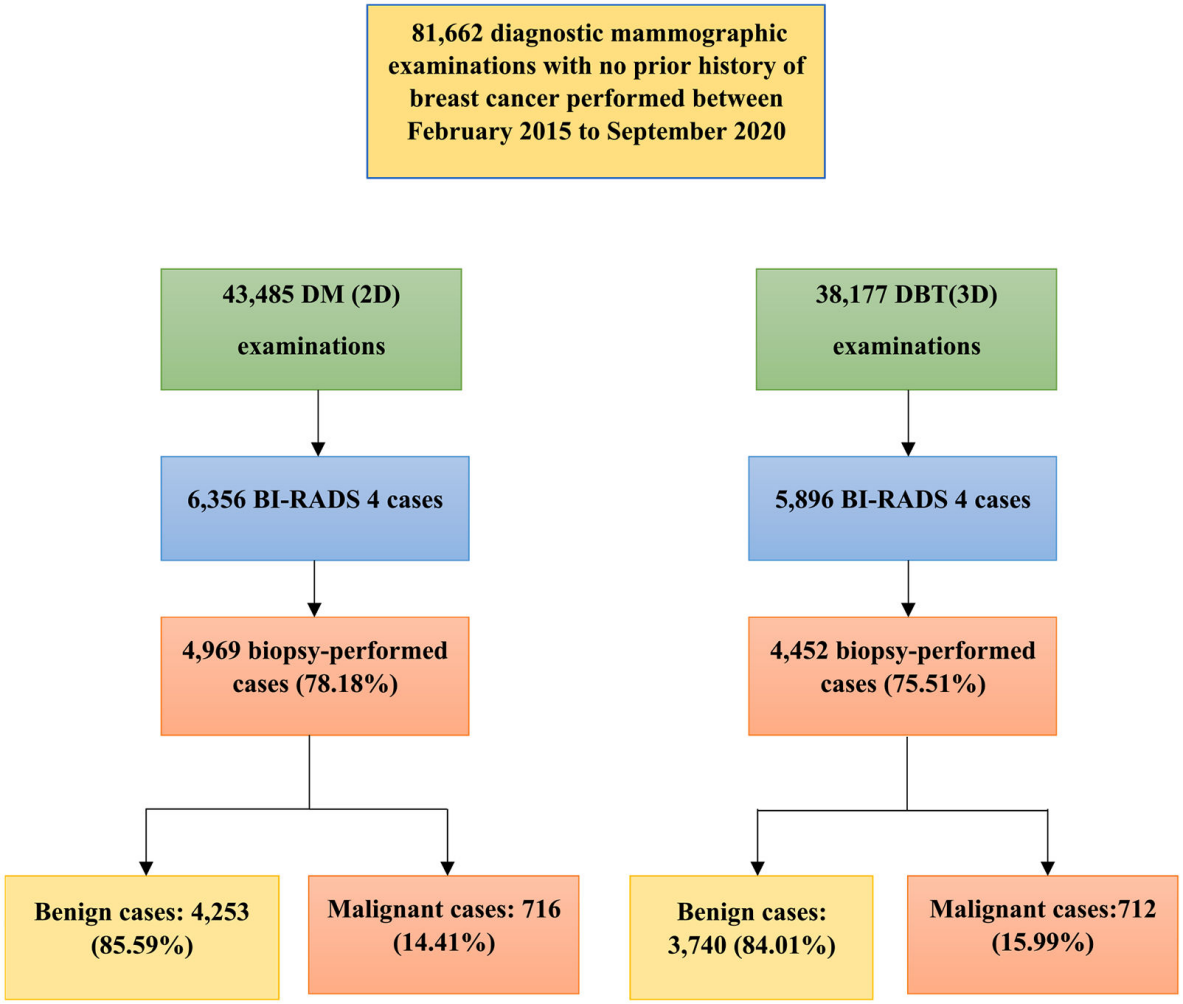
Flow diagram to show patient selection and the pathology including BI-RADS 4 results. DBT = Digital Breast tomosynthesis, 3D = 3 Dimensional, DM = Digital Mammography, 2D = 2 Dimensional, BI-RADS = Breast Imaging Reporting and Data System.

Within the over five-year period (February 2015 – September 2020), there were 6356 encounters (6020 unique patients) who had 2D mammography examinations and 5896 encounters (5637 unique patients) who had DBT examinations and were assessed as BI-RADS 4. Meanwhile, there were a total of 31,303 unique patients (43,485 encounters) and 29,538 unique patients (38,177 encounters) who had 2D and DBT mammography, respectively, performed across the nine hospitals of our health system (Fig. 1). Using Fisher’s exact test, the results show that the BI-RADS 4 assessed cases in DBT are slightly, yet significantly more than those in 2D mammography by 5.66%, p-value = 0.004591 (1.0566 95% CI: 1.0169–1.0977). There were only 35 (0.59%) cases among the DBT group who had a subsequent DBT examination after a 2D digital mammogram. In terms of descriptive statistics, a series of preliminary analyses were conducted on the patient factors (age, race, gender, menopausal status, and presence of prior mammogram), and tumor characteristics (staging, ER, PR, and HER2 status) in both modalities. We then analyzed the CDR and PPV3 of patients scanned by 2D mammography against patients scanned by DBT mammography.

### 3.1. Demographics and other patient factors

Demographics distribution and proportions in 2D and DBT populations were largely similar and comparable. The median age among BI-RADS 4 2D mammography cases was 55 years (20–95) and 54 years (22–99) in DBT cases. Most patients, 99.21% (6306) and 99.51% (5867), were women among the 2D and DBT BI-RADS 4 cohorts, respectively. In 2D, Caucasians were a majority with 4,321 cases (67.98%); followed by Blacks, 1,237 cases (19.46%); and Asians, 532 cases (8.37%). Racial distribution within the DBT group had a similar picture: Caucasians 4105 (69.62%), Blacks 1058 (17.94%), and Asians 550 (9.33%). This distribution of race in 2D mammography cases is significantly different from DBT cases *(P = 0.001195)*. Menopausal status proportions in both cohorts were similar as 3306 (52.01%) and 3088 (52.37%) were premenopausal in the 2D and DBT groups, respectively. A total of 4265 (67.10%) in 2D group and 3290 (55.80%) in DBT group had prior mammograms performed and the difference between them was significant (*p < 2e – 16*).

There were no statistically significant differences between the two modalities (2D vs. DBT mammography) concerning age (P = 0.7092), sex (0.05435), and menopausal status (P = 0.7031) in BI-RADS 4 category. Table 1 shows the summary of these patient characteristics.

Statistical distributions and p values (for significant differences in comparisons between 2D and DBT modalities) for the above parameters in the entire patient cohorts i.e., all BI-RADS categories are detailed in Supplementary Table 1.

### 3.2. Biopsy outcome, staging, and ER, PR, and HER2 status

Available biopsy outcome records showed that 14.41% (716/4969) in 2D mammography cases and 15.99% (712/4452) in DBT cases were malignant among the BI-RADS 4 cohort who had biopsy performed and results available. This difference was not significant based on Fisher’s test *(P = 0.0688)*. Difference in proportions of malignant BI-RADS 4 cases in the entire BI-RADS 4 population (biopsy or no biopsy) in 2D (11.26%) vs. DBT (12.07%) mammography was also found to be not statistically significant *(P = 0.2183)*. Distributions, proportions, and percentages of the malignant cases among biopsy cases as well as in the entire cohort (i.e., irrespective of whether they had biopsy or not) for the various BI-RADS groups and a combination of BI-RADS 1 through 5 i.e., without category 0 (inconclusive result) and 6 (known biopsy-proven malignant lesions) is shown in Supplementary Table 2.

A summary of tumor characteristics based on our available data is shown in Table 2. From the available tumor staging data, the distribution of staging based on 2D and DBT mammography in BI-RADS 4 cases had no significant difference using a Chi-squared test *(P = 0.0678)*. Similarly, when we analyzed available data on the ER, PR, and HER2 status results (i.e., positive/negative in ER and PR and positive/negative/equivocal in HER2), Fisher’s exact test showed no significant differences between 2D and DBT mammography (Table 3). Thus, tumor characteristics including staging, hormone receptor status (ER, PR), and growth-promoting protein (HER2) in our available data revealed no significant difference among BI-RADS 4 patients. For tumor characteristics among the entire patient cohort (all BI-RADS categories), between 2D and DBT modalities, please see Supplementary Table 3.

### 3.3. Cancer detection rate (CDR) and biopsy-derived positive predictive value (PPV3)

**Fig. 2.**
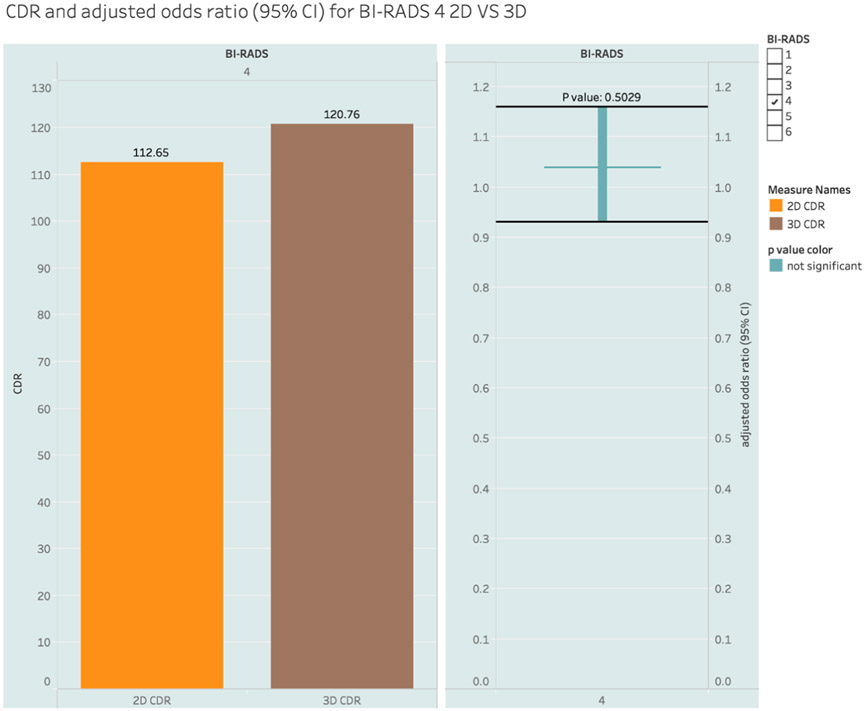
Cancer Detection Rate (CDR) of BI-RADS 4. CDR = Cancer Detection Rate, 3D = 3 Dimensional, 2D = 2 Dimensional, BI-RADS = Breast Imaging Reporting and Data System, CI – Confidence Interval.

**Fig. 3.**
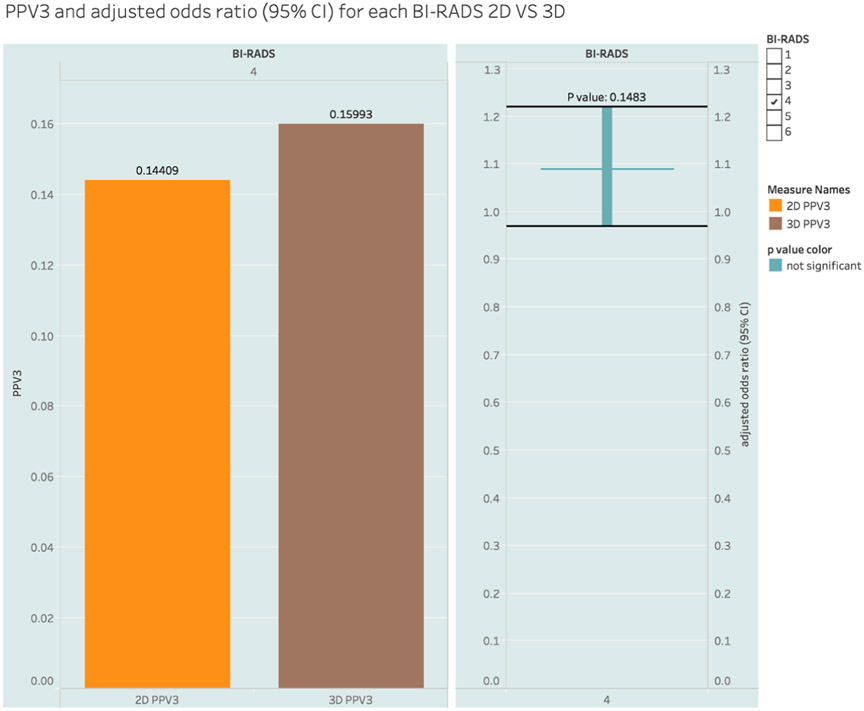
Biopsy-derived Positive Predictive Value (PPV3) of BI-RADS 4 PPV = Positive Predictive Value, 3D = 3 Dimensional, 2D = 2 Dimensional, BI-RADS = Breast Imaging Reporting and Data System, CI – Confidence Interval.

In the BI-RADS 4 category, the CDRs were 112.65 for 2D and 120.76 for DBT with logistic regression-adjusted odds ratio of 1.04 (0.93, 1.16), which was not significant *P = 0.5029*. Fig. 2 shows the CDR and the logistic regression-adjusted odds ratios for BI-RADS 4. Biopsy-derived positive predictive values (PPV3) among the BI-RADS 4 cohort were 14.41% (2D) and 15.99% (DBT) and again, this difference was not significant with an adjusted odds ratio of 1.09 (0.97, 1.22), *P = 0.1483*. Fig. 3 shows the PPV3 and the logistic regression-adjusted odds ratios for BI-RADS 4. In both cases, logistic regression was used to adjust the impact of all other variables.

Supplementary Figs. 1 and 2 detail the CDR and PPV3 and odd ratios of individual BI-RADS categories.

## 4. Discussion

In this study, we assessed the performance of 2D mammography in comparison with DBT (DBT tomograms only) among the cases that were given an initial assessment of BI-RADS 4 in diagnostic examinations. In our hospital system, DBT is not evaluated as a combination of synthetic 2D and DBT i.e., s2D + DBT. There have been some studies in this subject but often assessing entire dignostic cohorts i.e. with BI-RADS categories zero through six. Our study focuses on BI-RADS 4 designated lesions, which have a wide range of probability of malignancy and resultant overbiopy, thus making improved cancer detection highly desirable in this category. The advent of DBT has certainly resulted in the production of better images and better portrayal of massess, asymetries, and other anomalies by facilitating the separation of over-lapping structures common with 2D mammography [41]. Also, early studies have reported that DBT detects as much as 40 percent more cancers than digital (2D) mammograms in breast cancer screening examinations [42].

Nevertheless, our findings indicate that once the assessment is made as BI-RADS 4, biopsy outcomes were comparable for both DM and DBT as malignancy rates, cancer detection rates, and biopsy-derived positive predictive values were similar, such that differences were not statistically significant. A slightly deeper dive into granular data (histologic staging, hormone receptor and growth-promoting protein status) also showed similar performance. However, of note is that our results in BI-RADS 4 do not discount the fact that overall or when certain other BI-RADS categories are considered, DBT mammography might exhibit a better performance over 2D mammography.

It is worth pointing out that BI-RADS 4 demographic distributions including age, gender, and race were of similar patterns among 2D and DBT mammography cohorts in our data, and our data also mirrors established national statistics. For instance, Caucasians who have the highest overall incidence of breast cancer [43-45] are in the majority, followed by Blacks and then Asians in both cohorts. The racial distribution is significant between 2D and DBT mammography, yet there was no significant difference in the cancer detection rates. Despite histologic workup being recommended for all BI-RADS 4 patients, this is often not the case as seen in our health system where there is 24–25% unavailable biopsy data, probably due to loss in follow-up, change of hospital, etc.

The application of DBT has increased since 2012 after FDA’s approval the prior year. The modality, which was commonly used as a special application for supplementing 2D X-ray mammography in the case of suspicious findings initially, has became a standard of care in many organizations for breast evaluation with some centers including ours already using DBT as first line for mammography service. While some studies suggest that DBT detects cancers better than 2D DM and results in lower recall rates, especially when combined with actual or synthesized 2D images [46-49], one study suggests that there is no real advantage, reporting similarity in cancer detection rate [38].

Hofvind et al. suggested that DBT and 2D synthetic mammography (SM), recreated from tomosynthesis images, increased the detection rate of histologically favorable tumors compared with that attained from DM evaluation alone [47]. Li et al. concluded that DBT exhibits higher diagnostic accuracy for benign calcifications, dense breasts, and both premenopausal and postmenopausal women, but has no advantage in non-dense breasts and malignant calcification cases when compared to 2D digital mammography (DM) and recommended DBT for breast cancer evaluation in young women with dense breasts [48]. Bahl and colleagues in an earlier work demonstrated that overall cancer detection rates were similar in both the DM and DM + DBT cohorts, however the proportion of invasive cancers compared to in situ lesions as well as the PPV were significantly higher among the DM + DBT group compared to DM group [38].

Our more recent study comparing 2D digital mammography with mostly initial DBT (only 0.59% DM + DBT) suggests that breast cancer evaluation with DBT may not have much comparative advantage over DM in clinical practice when it comes to detecting cancers or improving the PPV3 for BI-RADS 4-assessed lesions. DBT does not reduce unnecessary biopsies associated with BI-RADS 4 and brings to the fore the need for tools that could help to mitigate the problem of unnecessary biopsies [50].

### 4.1. Limitations

This current work was done using data from a single large multi-hospital health system made up of nine hospitals and several clinics in Houston, Texas, including outpatient breast imaging centers, so we do not rule out the possibility of certain bias in the results, even though Houston is one of the most diverse cities in the United States. Further studies are warranted on this subject matter, especially across a wide geographical area and several health systems. Gray screening could be a limitation, however, we used mammograms designated strictly as diagnostic in terms of procedure name and physician notes, and the data was well validated. Another area that our data may engender limitation is the non-availability of BI-RADS 4 sub-categorizations i.e. 4a, 4b, and 4c. These are not reported in our hospital mammogram reports; it is noteworthy that this data is poorly documented, has low utilization prevalence, and is heterogenous in use across the US. Though we assessed performance in some granular data, i.e., histologic staging, hormone receptor and growth-promoting protein status, available data was limited. We could not look at the impact of tumor grading or breast fibrodensity and its higher risk, due to unavailability of this data. However, our data is homogeneous and demographic distributions including age and race are similar and balanced between the 2D and DBT cohorts. Also, an analysis of characteristics of histological types of cancers detected by DBT is a subject for future research. All of these could form the basis of future extension of this work to investigate if there are categories of patients that will benefit more from DBT modality evaluation if they are assigned as BI-RADS 4.

## 5. Conclusion

Among BI-RADS 4 assigned patients undergoing diagnostic examinations, DBT appears not to show statistically significant improvement to performance and sensitivity in assigning BI-RADS 4 cases when compared with DM as a slightly higher number (5.66%) of patients undergoing DBT mammography were assessed as BI-RADS 4 more often than those undergoing 2D mammography. Also, biopsy outcomes were comparable for both DM and DBT as malignancy rates, cancer detection rates, and biopsy-derived positive predictive values were similar, and differences were not statistically significant between modalities. Thus, DBT does not reduce unnecessary biopsies associated with BI-RADS 4 assessed patients. Our research findings would benefit from more extensive investigation, especially using data with an increased granularity and from a wider geographical coverage area.

## Supplementary Material

A comparative efficacy study of diagnostic DBT vs. DM supplementary materials

## Figures and Tables

**Table 1:** Summary of patient characteristics in BI-RADS 4 population (2D vs. 3D).

Patient characteristics	2D or digital mammography (n = 6356) no. (%)	3D or digital breast tomosynthesis (n = 5896) no. (%)	P-value
Age Median	55	54	0.7092
Mean	55.28	55.37	
Range	(20–95)	(22–99)	
Sex			0.05435
Female	6,306 (99.21)	5,867 (99.51)	
Male	50 (0.79)	29 (0.49)	
Race			0.001195
Asian	532 (8.37)	550 (9.33)	
Black	1,237 (19.46)	1,058 (17.94)	
Caucasian	4,321 (67.98)	4,105 (69.62)	
Others	266 (4.19)	183 (3.10)	
Menopausal status			0.7031
Pre	3,306 (52.01)	3,088 (52.37)	
Post	3,050 (47.99)	2,808 (47.63)	
Prior mammogram			<2e – 16
No	2,091 (32.90)	2,606 (44.20)	
Yes	4,265 (67.10)	3,290 (55.80)	

**Table 2:** Summary of tumor characteristics in BI-RADS 4 population (2D vs. 3D).

Tumor characteristics	2D or digital mammography no. (%)	3D or digital breast tomosynthesis no. (%)	P-value (Fishers’)
Staging	(n = 273)	(n = 274)	
Stage 0	68 (24.91)	44 (16.06)	0.03844
Stage I	137 (50.18)	162 (59.12)	0.2805
Stage II	39 (14.29)	36 (13.14)	0.8057
Stage III	6 (2.20)	4 (1.46)	0.7517
Stage IV	6 (2.20)	3 (1.09)	0.5043
Stage Unknown	17 (6.23)	25 (9.12)	0.2647
Estrogen Receptor (ER)	(n = 246)	(n = 272)	
Negative	41 (16.67)	45 (16.54)	1
Positive	205 (83.33)	227 (83.46)	1
Progesterone Receptor (PR)	(n = 242)	(n = 271)	
Negative	58 (23.97)	73 (26.94)	0.558
Positive	184 (76.03)	198 (73.06)	0.7869
HER2 Gene	(n = 165)	(n = 220)	
Negative	144 (87.27)	197 (89.55)	0.8806
Positive	20 (12.12)	18 (8.18)	0.3039
Equivocal	1 (0.61)	5 (2.27)	0.2472

## References

[1] Elmore JG, Armstrong K, Lehman CD, , Screening for breast cancer, Jama 293 (2005) 1245–1256. 15755947 10.1001/jama.293.10.1245 PMC3149836

[2] Dongola N: Mammography in Breast Cancer, in: Eby P. (Ed.), Drugs & Diseases – Radiology, Medscape, 2020.

[3] Race E. Breast Cancer Risk Factors, BreastCancer.org, 2021.

[4] Chlebowski RT, Chen Z, Anderson GL, Rohan T, Aragaki A, Lane D, Dolan NC, Paskett ED, McTiernan A, Hubbell FA, Adams-Campbell LL, Prentice R, Ethnicity and breast cancer: factors influencing differences in incidence and outcome, J. Natl. Cancer Inst 97 (6) (2005) 439–448. 15770008 10.1093/jnci/dji064

[5] Stapleton SM, Oseni TO, Bababekov YJ, Hung Y-C, Chang DC, Race/ethnicity and age distribution of breast cancer diagnosis in the united states, JAMA Surg. 153 (6) (2018) 594, 10.1001/jamasurg.2018.0035. 29516087 PMC5875337

[6] Balleyguier C, Ayadi S, Van Nguyen K, Vanel D, Dromain C, Sigal R, BIRADS^™^ classification in mammography, Euro. J. Radiol 61 (2) (2007) 192–194. 10.1016/j.ejrad.2006.08.033 17164080

[7] D’Orsi C, Bassett L, Feig S, Breast imaging reporting and data system (BI-RADS), in: Breast imaging atlas, 4th ed. American College of Radiology, Reston, 2018.

[8] Elezaby M, Li G, Bhargavan-Chatfield M, Burnside ES, DeMartini WB, ACR BI-RADS assessment category 4 subdivisions in diagnostic mammography: utilization and outcomes in the national mammography database, Radiology 287 (2) (2018) 416–422. 29315061 10.1148/radiol.2017170770 PMC6413875

[9] Weaver DL, Rosenberg RD, Barlow WE, Ichikawa L, Carney PA, Kerlikowske K, Buist DSM, Geller BM, Key CR, Maygarden SJ, Ballard-Barbash R, Pathologic findings from the Breast Cancer Surveillance Consortium: population-based outcomes in women undergoing biopsy after screening mammography, Cancer 106 (4) (2006) 732–742. 16411214 10.1002/cncr.21652

[10] Bent CK, Bassett LW, D’Orsi CJ, Sayre JW, The positive predictive value of BI-RADS microcalcification descriptors and final assessment categories, AJR Am. J. Roentgenol 194 (5) (2010) 1378–1383. 20410428 10.2214/AJR.09.3423

[11] Dahabreh IJ, Wieland LS, Adam GP, Halladay C, Lau J, Trikalinos T, A: core needle and open surgical biopsy for diagnosis of breast lesions: an update to the 2009 report, 2014. 25275206

[12] Bruening W, Fontanarosa J, Tipton K, , Systematic review: comparative effectiveness of core-needle and open surgical biopsy to diagnose breast lesions, Ann. Int. Med 152 (2010) 238–246. 20008742 10.7326/0003-4819-152-1-201001050-00190

[13] Luo W-Q, Huang Q-X, Huang X-W, Hu H-T, Zeng F-Q, Wang W, Predicting breast cancer in breast imaging reporting and data system (BI-RADS) ultrasound category 4 or 5 lesions: a nomogram combining radiomics and BI-RADS, Sci. Rep 9 (1) (2019), 10.1038/s41598-019-48488-4. PMC6695380 31417138

[14] Liu G, Zhang M-K, He Y, Liu Y, Li X-R, Wang Z-L, BI-RADS 4 breast lesions: could multi-mode ultrasound be helpful for their diagnosis? Gland. Surg 8 (3) (2019) 258–270. 31328105 10.21037/gs.2019.05.01 PMC6606479

[15] Strigel RM, Burnside ES, Elezaby M, Fowler AM, Kelcz F, Salkowski LR, DeMartini WB, Utility of BI-RADS assessment category 4 subdivisions for screening breast MRI, AJR Am. J. Roentgenol 208 (6) (2017) 1392–1399. 28792802 10.2214/AJR.16.16730 PMC5600516

[16] Halladay JR, Yankaskas BC, Bowling JM, Alexander C, Positive predictive value of mammography: comparison of interpretations of screening and diagnostic images by the same radiologist and by different radiologists, AJR Am. J. Roentgenol 195 (3) (2010) 782–785. 20729460 10.2214/AJR.09.2955 PMC4451561

[17] van Luijt PA, Fracheboud J, Heijnsdijk EAM, den Heeten GJ, de Koning HJ, Nation-wide data on screening performance during the transition to digital mammography: observations in 6 million screens, Eur. J. Cancer 49 (16) (2013) 3517–3525. 23871248 10.1016/j.ejca.2013.06.020

[18] Lehman CD, Lee CI, Loving VA, Portillo MS, Peacock S, DeMartini WB, Accuracy and value of breast ultrasound for primary imaging evaluation of symptomatic women 30–39 years of age, AJR Am. J. Roentgenol 199 (5) (2012) 1169–1177. 23096195 10.2214/AJR.12.8842

[19] Flowers CI, O’Donoghue C, Moore D, Goss A, Kim D, Kim J-H, Elias SG, Fridland J, Esserman LJ, Reducing false-positive biopsies: a pilot study to reduce benign biopsy rates for BI-RADS 4A/B assessments through testing risk stratification and new thresholds for intervention, Breast Cancer Res. Treat 139 (3) (2013) 769–777. 23764994 10.1007/s10549-013-2576-0 PMC3695318

[20] D’Orsi CJ, The clinically relevant breast imaging audit, J. Breast Imag 2 (1) (2020) 2–6. 10.1093/jbi/wbz080 38424999

[21] Radiology ACo: The Basic Clinically Relevant Audit, 2013.

[22] Ong MS, Mandl KD, National expenditure for false-positive mammograms and breast cancer overdiagnoses estimated at $4 billion a year, Health Aff. (Millwood) 34 (2015) 576–583. 25847639 10.1377/hlthaff.2014.1087

[23] Zeeshan M, Salam B, Khalid QSB, , Diagnostic accuracy of digital mammography in the detection of breast cancer, Cureus 10 (2018) e2448. 29888152 10.7759/cureus.2448 PMC5991925

[24] Johnson K, Sarma D, Hwang ES, Lobular breast cancer series: imaging, Breast Cancer Res. 17 (2015) 94. 26163296 10.1186/s13058-015-0605-0 PMC4499185

[25] Joy J, Penhoet E, Petitti D, Institute of Medicine (US) and National Research Council (US) Committee on New Approaches to Early Detection and Diagnosis of Breast Cancer. Saving Women’s Lives: Strategies for Improving Breast Cancer Detection and Diagnosis, 2005. 20669447

[26] Heywang-Köbrunner SH, Jänsch A, Hacker A, Weinand S, Vogelmann T, Digital breast tomosynthesis (DBT) plus synthesised two-dimensional mammography (s2D) in breast cancer screening is associated with higher cancer detection and lower recalls compared to digital mammography (DM) alone: results of a systematic review and meta-analysis, Eur. Radiol 32 (4) (2022) 2301–2312. 34694451 10.1007/s00330-021-08308-8 PMC8921114

[27] Conant EF, Barlow WE, Herschorn SD, Weaver DL, Beaber EF, Tosteson ANA, Haas JS, Lowry KP, Stout NK, Trentham-Dietz A, diFlorio-Alexander RM, Li CI, Schnall MD, Onega T, Sprague BL, Association of digital breast tomosynthesis vs digital mammography with cancer detection and recall rates by age and breast density, JAMA Oncol. 5 (5) (2019) 635, 10.1001/jamaoncol.2018.7078. 30816931 PMC6512257

[28] Neighmond P: When It’s Time For A Mammogram, Should You Ask For 3D?, Shots Health News From NPR: Your Health. Houston, TX, NPR Houston Public Media, 2019.

[29] Bernardi D, Ciatto S, Pellegrini M, Tuttobene P, Fanto’ C, Valentini M, Michele SD, Peterlongo P, Houssami N, Prospective study of breast tomosynthesis as a triage to assessment in screening, Breast Cancer Res. Treatment 133 (1) (2012) 267–271. 10.1007/s10549-012-1959-y 22270938

[30] Friedewald SM, Rafferty EA, Rose SL, Durand MA, Plecha DM, Greenberg JS, Hayes MK, Copit DS, Carlson KL, Cink TM, Barke LD, Greer LN, Miller DP, Conant EF, Breast cancer screening using tomosynthesis in combination with digital mammography, Jama-J. Am. Med. Assoc 311 (24) (2014) 2499, 10.1001/jama.2014.6095. 25058084

[31] Greenberg JS, Javitt MC, Katzen J, Michael S, Holland AE, Clinical performance metrics of 3D digital breast tomosynthesis compared with 2D digital mammography for breast cancer screening in community practice, AJR Am. J. Roentgenol 203 (3) (2014) 687–693. 24918774 10.2214/AJR.14.12642

[32] Lang K, Andersson I, Rosso A, , Performance of one-view breast tomosynthesis as a stand-alone breast cancer screening modality: results from the Malmo Breast Tomosynthesis Screening Trial, a population-based study, Euro. Radiol 26 (2016) 184–190. 10.1007/s00330-015-3803-3 PMC4666282 25929946

[33] Rose SL, Tidwell AL, Bujnoch LJ, Kushwaha AC, Nordmann AS, Sexton R, Implementation of breast tomosynthesis in a routine screening practice: an observational study, AJR Am. J. Roentgenol 200 (6) (2013) 1401–1408. 23701081 10.2214/AJR.12.9672

[34] Skaane P, Bandos AI, Gullien R, Eben EB, Ekseth U, Haakenaasen U, Izadi M, Jebsen IN, Jahr G, Krager M, Niklason LT, Hofvind S, Gur D, Comparison of digital mammography alone and digital mammography plus tomosynthesis in a population-based screening program, Radiology 267 (1) (2013) 47–56. 23297332 10.1148/radiol.12121373

[35] Yun SJ, Ryu C-W, Rhee SJ, Ryu JK, Oh JY, Benefit of adding digital breast tomosynthesis to digital mammography for breast cancer screening focused on cancer characteristics: a meta-analysis, Breast Cancer Res. Treat 164 (3) (2017) 557–569. 28516226 10.1007/s10549-017-4298-1

[36] Hodgson R, Heywang-Köbrunner SH, Harvey SC, Edwards M, Shaikh J, Arber M, Glanville J, Systematic review of 3D mammography for breast cancer screening, Breast 27 (2016) 52–61. 27212700 10.1016/j.breast.2016.01.002

[37] Houssami N, Miglioretti DL, Digital breast tomosynthesis: a brave new world of mammography screening, JAMA Oncol. 2 (6) (2016) 725, 10.1001/jamaoncol.2015.5569. 26892954

[38] Bahl M, Mercaldo S, Vijapura CA, McCarthy AM, Lehman CD, Comparison of performance metrics with digital 2D versus tomosynthesis mammography in the diagnostic setting, Eur. Radiol 29 (2) (2019) 477–484. 29967957 10.1007/s00330-018-5596-7

[39] Puppala M, He T, Chen S, Ogunti R, Yu X, Li F, Jackson R, Wong STC, METEOR: an enterprise health informatics environment to support evidence-based medicine, IEEE Trans. Biomed. Eng 62 (12) (2015) 2776–2786. 26126271 10.1109/TBME.2015.2450181

[40] Team RC: R: A language and environment for statistical computing.

[41] Ali EA, Adel L, Study of role of digital breast tomosynthesis over digital mammography in the assessment of BIRADS 3 breast lesions, Egypt. J. Radiol. Nucl. Med 50 (2019) 48.

[42] Conant EF, Beaber EF, Sprague BL, Herschorn SD, Weaver DL, Onega T, Tosteson ANA, McCarthy AM, Poplack SP, Haas JS, Armstrong K, Schnall MD, Barlow WE, Breast cancer screening using tomosynthesis in combination with digital mammography compared to digital mammography alone: a cohort study within the PROSPR consortium, Breast Cancer Res. Treatment 156 (1) (2016) 109–116. 10.1007/s10549-016-3695-1 PMC5536249 26931450

[43] Howlader N, Noone A, Krapcho M , SEER Cancer Statistics Review, 1975–2016. Bethesda, MD: National Cancer Institute, 2019.

[44] Miller BA, Chu KC, Hankey BF, Ries LAG, Cancer incidence and mortality patterns among specific Asian and Pacific Islander populations in the US, Cancer Causes Control 19 (3) (2008) 227–256. 18066673 10.1007/s10552-007-9088-3 PMC2268721

[45] ACS: Breast Cancer Facts & Figures 2019–2020. Atlanta, American Cancer Society, Inc., 2019.

[46] Aase HS, Holen ÅS, Pedersen K, Houssami N, Haldorsen IS, Sebuødegård S, Hanestad B, Hofvind S, A randomized controlled trial of digital breast tomosynthesis versus digital mammography in population-based screening in Bergen: interim analysis of performance indicators from the To-Be trial, Eur. Radiol 29 (3) (2019) 1175–1186. 30159620 10.1007/s00330-018-5690-x PMC6510877

[47] Hofvind S, Hovda T, Holen ÅS, Lee CI, Albertsen J, Bjørndal H, Brandal SHB, Gullien R, Lømo J, Park D, Romundstad L, Suhrke P, Vigeland E, Skaane P, Digital breast tomosynthesis and synthetic 2D mammography versus digital mammography: evaluation in a population-based screening program, Radiology 287 (3) (2018) 787–794. 29494322 10.1148/radiol.2018171361

[48] Li J, Zhang H, Jiang H, Guo X, Zhang Y, Qi D, Guan J, Liu Z, Wu E, Luo S, Diagnostic performance of digital breast tomosynthesis for breast suspicious calcifications from various populations: a comparison with full-field digital mammography, Comput. Struct. Biotechnol. J 17 (2019) 82–89. 30622686 10.1016/j.csbj.2018.12.004 PMC6317146

[49] Mumin NA, Rahmat K, Fadzli F, Ramli MT, Westerhout CJ, Ramli N, Rozalli FI, Ng KH, Diagnostic efficacy of synthesized 2D digital breast tomosynthesis in multi-ethnic Malaysian population, Sci Rep 9 (1) (2019), 10.1038/s41598-018-37451-4. PMC6365555 30728394

[50] He T, Puppala M, Ezeana CF, Huang Y-S, Chou P-H, Yu X, Chen S, Wang L, Yin Z, Danforth RL, Ensor J, Chang J, Patel T, Wong STC, A deep learning-based decision support tool for precision risk assessment of breast cancer, JCO Clin. Cancer Inform (3) (2019) 1–12, 10.1200/CCI.18.00121. PMC10445790 31141423

