# Supplementary material for: A comparative efficacy study of diagnostic digital breast tomosynthesis and digital mammography in BI-RADS 4 breast cancer diagnosis
Source: Eur J Radiol. Author manuscript; Available in PMC 2023 Jul 14. (PMC10347302; doi:10.1016/j.ejrad.2022.110361)
Supplement: A comparative efficacy study of diagnostic DBT vs. DM supplementary materials [file NIHMS1908619-supplement-A_comparative_efficacy_study_of_diagnostic_DBT_vs__DM_supplementary_materials.docx]

**A Comparative Efficacy Study of Diagnostic Digital Breast Tomosynthesis and Digital Mammography in BI-RADS 4 Breast Cancer Diagnosis.**

**SUPPLEMENTAL FIGURES AND TABLES**

**Supplemental Figure 1: Cancer Detection Rate (CDR) of BI-RADS categories 1 through 5.**

CDR = Cancer Detection Rate, 3D = 3 Dimensional, 2D = 2 Dimensional, BI-RADS = Breast Imaging Reporting and Data System, , CI - Confidence Interval.


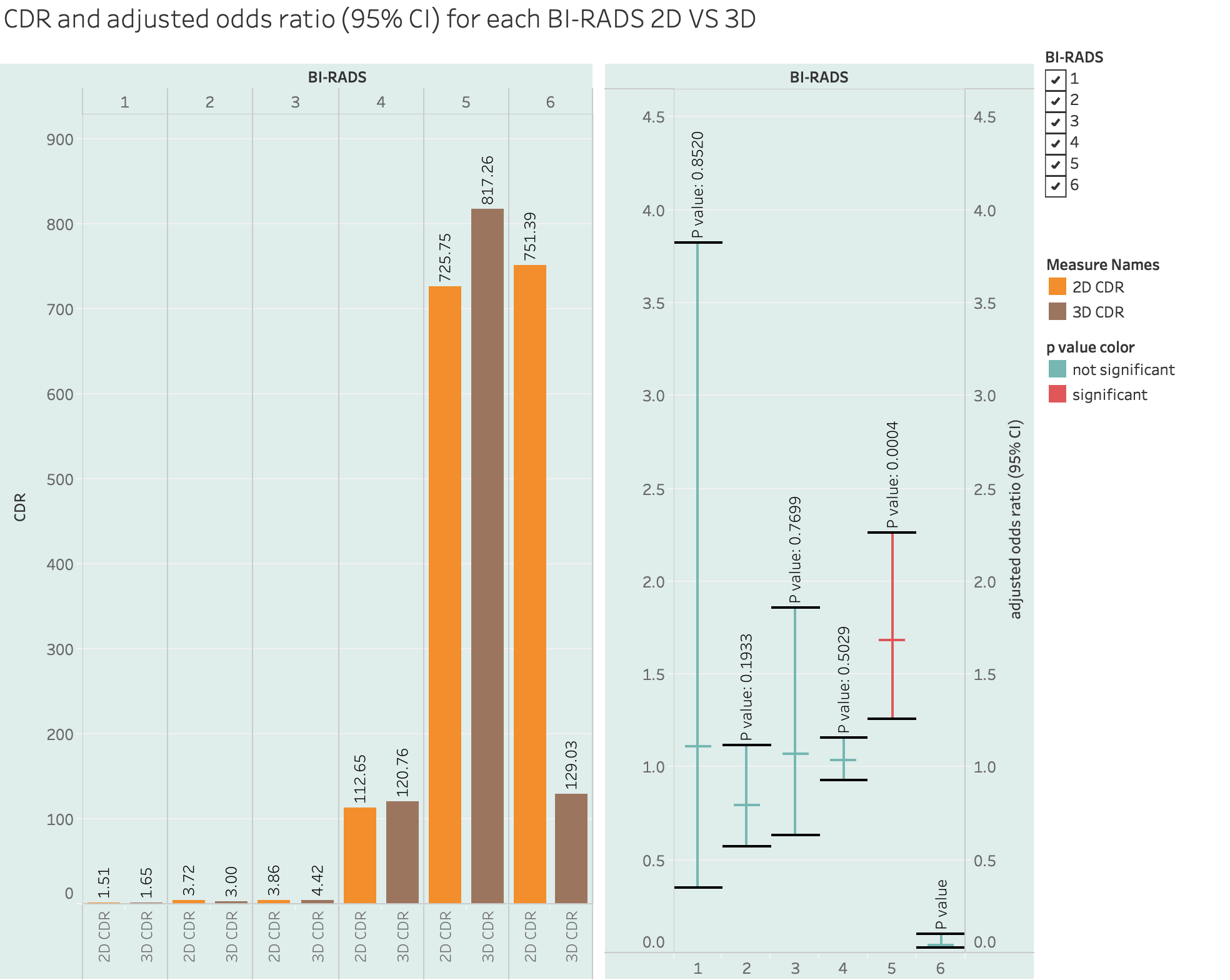


**Supplemental Figure 2: Biopsy-derived Positive Predictive Value (PPV3) of BI-RADS categories 1 through 5.**

PPV = Positive Predictive Value, 3D = 3 Dimensional, 2D = 2 Dimensional, BI-RADS = Breast Imaging Reporting and Data System, CI - Confidence Interval.


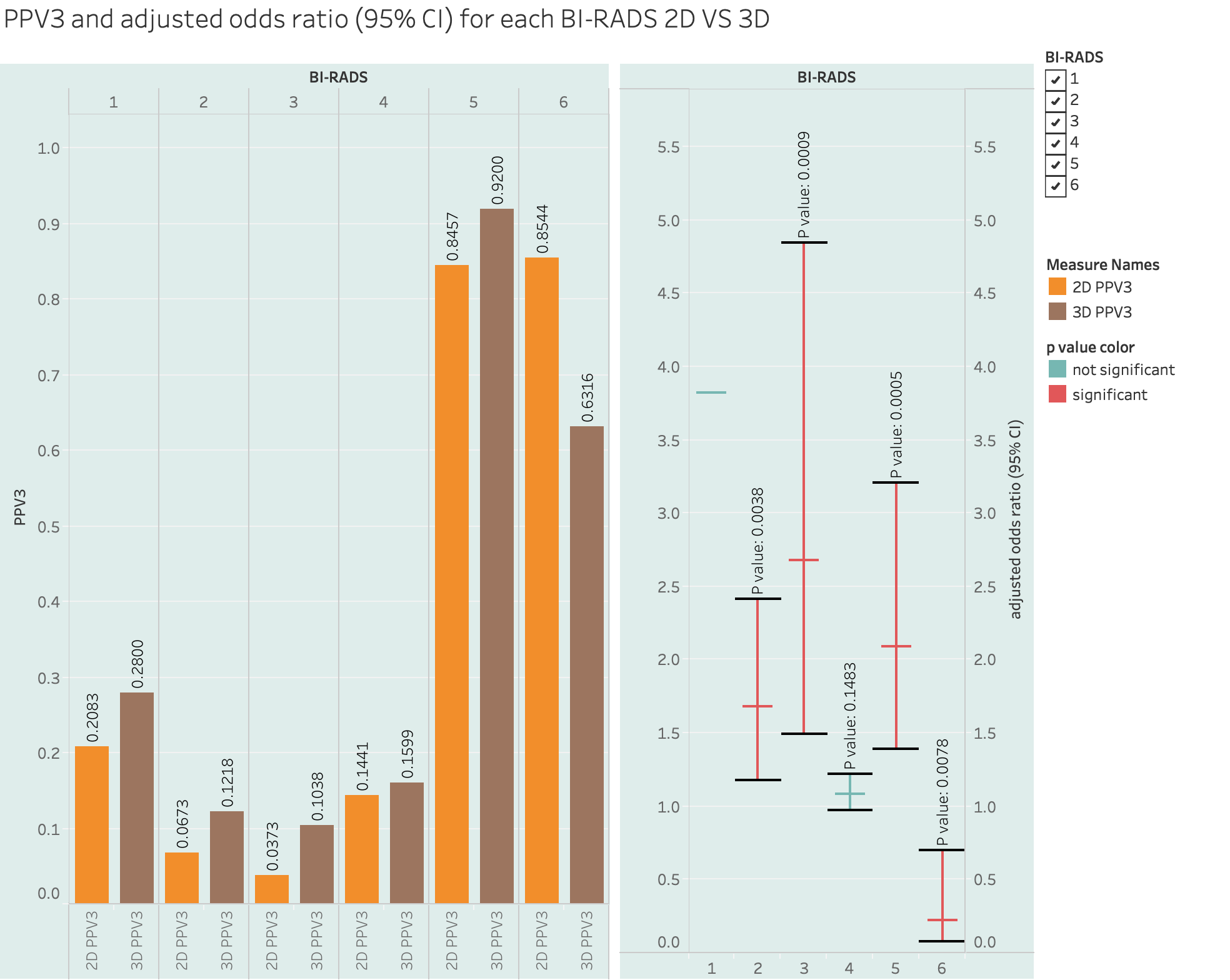


**Supplemental Table 1: Summary of patient characteristics in complete mammography population (2D vs. 3D).**

| Patient characteristics | 2D or Digital Mammography (n = 43, 485) No. (%) | 3D or Digital Breast Tomosynthesis (n = 38, 177) No. (%) | P-value |
| --- | --- | --- | --- |
|  |  |  |  |
| Age Median  Mean  (Range) | 55  55.65  (15 - 99) | 55  55.89  (18 - 99) | 0.008457 |
| Sex |  |  | <2.2e-16 |
| Female | 42,685 (98.16) | 37,799 (99.00) |  |
| Male | 800 (1.84) | 378 (1.00) |  |
| Race |  |  | <2.2e-16 |
| Asian | 3,236 (7.44) | 2,945 (7.71) |  |
| Black | 7,665 (17.63) | 6,266 (16.41) |  |
| Caucasian | 30,566 (70.29) | 27,641 (72.40) |  |
| Others | 2,018 (4.64) | 1,325 (3.48) |  |
| Marital Status |  |  | 4.34e-14 |
| Married | 30,240 (69.54) | 27,442 (71.88) |  |
| Unmarried | 12,953 (29.79) | 10,553 (27.64) |  |
| Unknown | 292 (0.67) | 182 (0.48) |  |
| Menopausal Status |  |  | 0.0471 |
| Pre | 21,265 (48.90) | 18,936 (49.60) |  |
| Post | 22,220 (51.10) | 19,241 (50.40) |  |
| Prior Mammogram |  |  | <2e-16 |
| No | 10,934 (25.14) | 17,366 (45.49) |  |
| Yes | 32,551 (74.86) | 20,811 (54.51) |  |

**Supplemental Table 2: Malignancy rates among BI-RADS categories 2D vs. 3D**

|  | **2D** | | | | | **3D** | | | | |  |  | |
| --- | --- | --- | --- | --- | --- | --- | --- | --- | --- | --- | --- | --- | --- |
| **BI-RADS** | **No Biopsy** | **B** | **M** | **M/Sum** | **M/All Biopsy cases** | **No Biopsy** | **B** | **M** | **M/sum** | **M/All biopsy cases** | **P value (M/sum)** | **P value (M/All biopsy)** |  |
| **1** | **3291** | **19** | **5** | **0.0015** | **0.2083** | **4227** | **18** | **7** | **0.0016** | **0.28** | **1** | **0.7529** |  |
| **2** | **23129** | **1262** | **91** | **0.0037** | **0.0673** | **20179** | **447** | **62** | **0.0030** | **0.1218** | **0.1946** | **0.0007444** |  |
| **3** | **6498** | **723** | **28** | **0.0039** | **0.0373** | **5852** | **233** | **27** | **0.0044** | **0.1038** | **0.6847** | **0.0004469** |  |
| **4** | **1387** | **4253** | **716** | **0.1126** | **0.1441** | **1444** | **3740** | **712** | **0.1208** | **0.1599** | **0.2183** | **0.06882** |  |
| **5** | **76** | **71** | **389** | **0.7257** | **0.8457** | **66** | **42** | **483** | **0.8173** | **0.92** | **0.2054** | **0.3755** |  |
| **Totals** | **34381** | **6328** | **1229** | **0.0293** | **0.1626** | **31768** | **4480** | **1291** | **0.0344** | **0.2237** | **7.742e-05** | **2.717e-13** |  |

**Supplemental Table 3: Summary of tumor characteristics in complete mammographic population (2D vs. 3D)**

| Tumor Characteristics | 2D or Digital Mammography No. (%) | 3D or Digital Breast Tomosynthesis No. (%) | P-value |
| --- | --- | --- | --- |
|  |  |  |  |
| Staging | (n = 682) | (n = 534) |  |
| Stage 0 | 94 (13.78) | 49 (9.18) | 0.03182 |
| Stage I | 320 (46.92) | 273 (51.12) | 0.4202 |
| Stage II | 156 (22.87) | 113 (21.16) | 0.5872 |
| Stage III | 51 (7.48) | 40 (7.49) | 1 |
| Stage IV | 20 (2.93) | 18 (3.37) | 0.7409 |
| Stage Unknown | 41 (6.01) | 41 (7.68) | 0.3024 |
| Estrogen Receptor (ER) | (n = 617) | (n = 522) |  |
| Negative | 133 (21.56) | 108 (20.69) | 0.831 |
| Positive | 484 (78.44) | 414 (79.31) | 0.9287 |
| Progesterone Receptor (PR) | (n = 612) | (n = 521) |  |
| Negative | 193 (31.54) | 157 (30.13) | 0.759 |
| Positive | 419 (68.46) | 364 (69.87) | 0.8521 |
| HER2 Gene | (n = 506) | (n = 500) |  |
| Negative | 441 (87.15) | 387 (83.77) | 0.7045 |
| Positive | 52 (10.28) | 63 (13.64) | 0.1674 |
| Equivocal | 13 (2.57) | 12 (2.60) | 1 |
